# Comparison of Baseline Wander Removal Techniques considering the Preservation of ST Changes in the Ischemic ECG: A Simulation Study

**DOI:** 10.1155/2017/9295029

**Published:** 2017-03-08

**Authors:** Gustavo Lenis, Nicolas Pilia, Axel Loewe, Walther H. W. Schulze, Olaf Dössel

**Affiliations:** ^1^Karlsruhe Institute of Technology (KIT), Institute of Biomedical Engineering (IBT), Fritz-Haber-Weg 1, 76131 Karlsruhe, Germany; ^2^EP Solutions SA, Rue Galilée 7, 1400 Yverdon-les-Bains, Switzerland

## Abstract

The most important ECG marker for the diagnosis of ischemia or infarction is a change in the ST segment. Baseline wander is a typical artifact that corrupts the recorded ECG and can hinder the correct diagnosis of such diseases. For the purpose of finding the best suited filter for the removal of baseline wander, the ground truth about the ST change prior to the corrupting artifact and the subsequent filtering process is needed. In order to create the desired reference, we used a large simulation study that allowed us to represent the ischemic heart at a multiscale level from the cardiac myocyte to the surface ECG. We also created a realistic model of baseline wander to evaluate five filtering techniques commonly used in literature. In the simulation study, we included a total of 5.5 million signals coming from 765 electrophysiological setups. We found that the best performing method was the wavelet-based baseline cancellation. However, for medical applications, the Butterworth high-pass filter is the better choice because it is computationally cheap and almost as accurate. Even though all methods modify the ST segment up to some extent, they were all proved to be better than leaving baseline wander unfiltered.

## 1. Introduction

**Figure 1:**
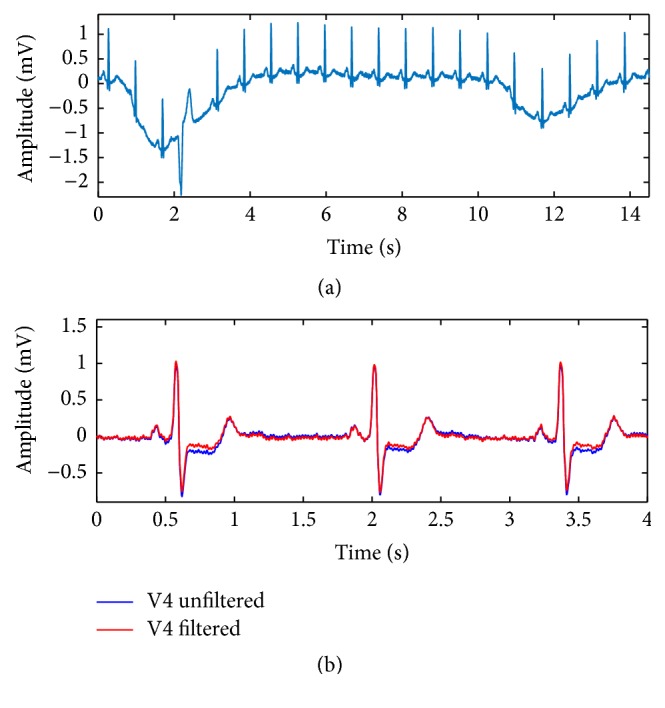
(a) ECG recording corrupted by baseline wander. The removal of this artifact is necessary when diagnosing a change in the ST segment. Yet, the filtering process can modify the signal as seen in (b). (b) ECG recording with a clear ST depression before (blue) and after (red) high-pass filtering. The ST depression is reduced because of too strong filtering. The signals presented in this figure were retrieved from the Physionet database [20].

Notorious changes in the ST segment (elevation or depression) are the most important ECG marker when dealing with acute coronary syndrome caused by ischemia or mycardial infarction [1]. Baseline wander is a low frequency artifact in the ECG that arises from breathing, electrically charged electrodes, or subject movement and can hinder the detection of these ST changes because of the varying electrical isoline (Figure 1(a)). The guidelines for diagnosis of myocardial ischemia are based on specific, small changes in the ST segment. Therefore, even minor fluctuations in baseline can lead to the decision if a patient is classified as STEMI or non-STEMI and thus influence the therapeutic approach dramatically [1]. This decision is often taken in a preclinical, out-of-hospital scenario with little or no infrastructural noise control. A further situation, where baseline wander becomes critical for the diagnosis of an ST change, is cardiac stress testing [2]. The idea behind this procedure is to measure the response of the heart to exercise on a treadmill and to study coronary circulation in comparison to resting conditions. This test should be terminated immediately if, among other criteria, significant ST changes appear in the ECG [3]. However, the observation of those changes becomes difficult if the ECG baseline is not constant [4].

Hence, baseline drift must be removed and, as a matter of fact, it is a standard signal processing step in many devices or postprocessing algorithms [5–7]. The majority of baseline wander removal techniques have in common that they cancel the low frequency components of the ECG signal. By doing so, they can also alter the ischemia-induced changes in the ST segment and compromise its clinical relevance [8]. An example of this kind of behavior can be seen in Figure 1(b). A recorded ECG with a clear ST depression is filtered with a high-pass filter diminishing that depression. This is the reason why recommendations have been published stating that high-pass filters with a cut-off frequency not higher than 0.05 Hz should be used for these kind of applications [9]. Yet, it is also known that baseline wander may be located at higher frequencies mandating more intrusive filters with higher cut-off frequencies [10, 11]. Thus, finding the filters that are capable of removing baseline wander without compromising ST changes is a relevant task.

Even though some interesting approaches to address this question have been reported in literature [12–14], it is not trivial to perform such a study using only baseline corrupted ECG recordings. Since the ground truth (artifact free signal) is not known, it is hard to objectively evaluate filter performance based on modifications of the ST segment. Furthermore, they are prone to detection errors from the automatic signal processing software, because some of the methods require, for example, a precise annotation of the R peak or P wave [15]. On the other hand, synthetic signals have also been used for these applications, but they might not be realistic enough to reproduce true changes of the ST segment [16]. In order to overcome this limitation, in silico studies can be used. The simulation of cardiac electrophysiology allows us to recreate ischemia at a multiscale level. We can adjust the models that govern the action potential (AP) in the cardiac myocyte to represent ischemia-induced change, let the electrical depolarization and subsequent repolarization propagate in the heart, and generate the body surface potential map on the chest. We can then place electrodes on the torso and extract the simulated 12-lead ECG [17, 18]. With this approach, we have the advantage of being able to locate the most relevant fiducial points (e.g., QRS complex or ST segment) in the simulated ECG and are thus capable of performing an analysis that is free of detection errors.

By adding baseline wander to the ECG, we reproduce the signals in a controlled environment that would have been recorded in real life. Since the ground truth is also known, the performance of the filters can be studied. Moreover, the simulation software allows varying the patient geometry, the location, and size of the ischemia in the heart and the electrical properties of the model. This leads to a large number of possible ECG signals and ST changes.

In this study, we address the question of the best suited technique for baseline removal without compromising ST changes. For that purpose, we use a diverse database of simulated ECGs with a state-of-the-art electrophysiological model, add synthetic baseline wander, and compare the performance of five manually tuned filters commonly used in literature. Preliminary results on a smaller study including fewer filters and less signals were presented at the national conference of biomedical engineering in Germany [19].

## 2. Materials and Methods

### 2.1. Generation of Simulated Data

The database containing the 3 × 255 = 765 electrophysiological setups used in this research project was originally created in our group with the aim of studying optimal electrode placement to detect ischemia in the ECG [21, 22]. The data set was created using three different subjects. Their geometries were obtained by segmenting the Visible Man dataset and two other magnetic resonance images. Distinct tissue classes were used for the main organs including the ventricles, skeletal muscle, fat, blood, lung, liver, and spleen. Fiber orientation in the ventricles was introduced using a rule-based approach [23]. The voxel meshes of the three subjects were created with an isotropic side length no greater than 0.5 mm.

A cellular automaton was chosen to perform the ischemia simulations [24]. This is a computationally cheap method based on predefined rules. In all simulations, the electrical depolarization wave originates from the Purkinje muscle junctions. It then propagates by sequentially activating neighboring voxels and triggering an AP in each voxel. The repolarization scheme is thus dependent on the AP corresponding to every region of the heart. Transmural heterogeneity and anisotropic tissue conductivities inside the ventricular walls were included in the model [25].

The AP in each voxel was obtained from a monodomain simulation carried out using the software acCELLerate and utilizing the Ten Tusscher cell model with a basic cycle length of 60 beats per minute [26–28]. The model parameters were adjusted to differentiate healthy tissue from ischemic regions [29, 30]. The latter were placed in all 17 AHA segments in the left ventricle and varied in size to include subendocardial and transmural scenarios [31].

From every simulation, a QRS complex, an ST segment, and a T wave were obtained. The QT interval was equal to 400 ms in all simulations. The sampling frequency of the simulated signals was 500 Hz, but upsampling to *f*_*s*_ = 512 Hz was carried out to facilitate wavelet-based filtering.

**Figure 2:**
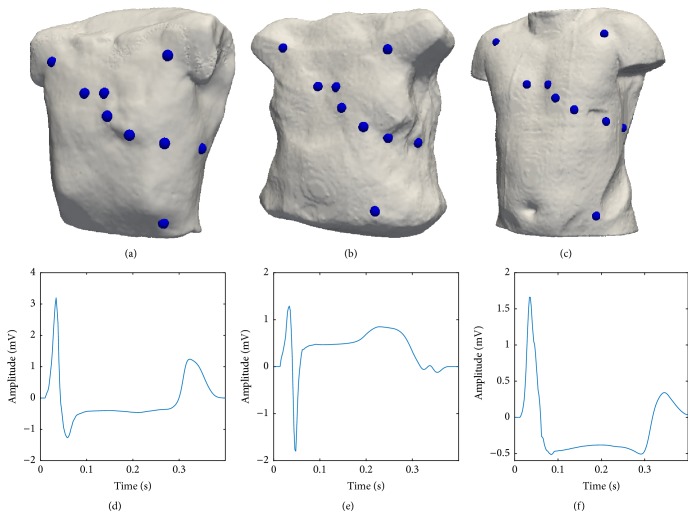
Demonstration of the three torso models and one example per torso of an ischemic ECG. (a), (b), and (c) torso model and electrode placement of the first, second, and third geometry used in the study. (d) Simulated ECG (Wilson lead V5) with ST depression using the torso model displayed in (a). This ECG is the result of a transmural ischemia with a radius of 20 mm and located in AHA segment 14. (e) Simulated ECG (Einthoven lead I) with ST elevation using the torso model displayed in (b). This ECG is the result of a transmural ischemia with a radius of 25 mm in AHA segment 13. (f) Simulated ECG (Einthoven lead II) with ST depression using the third torso model displayed in (c). This ECG is the result of a transmural ischemia with a radius of 20 mm in AHA segment 5.

Finally, a quasiperiodical extension was carried out to create an ECG with a fixed length of 100 s (51200 samples). In order to make the signal more realistic and recreate heart rate variability (HRV), variable RR intervals were added. RR intervals were modeled with a Gaussian distribution having an expected value of 1 s and a standard deviation of 50 ms. These parameters are in accordance with normal short term HRV values reported in literature [32].  Figure 2 shows the geometry of all three torso models and the electrode placement. A simulated signal corresponding to different ECG leads can be seen for each geometry. A clear ST change coming from the ischemia is also present.

### 2.2. Signal Processing Methods

#### 2.2.1. Modeling Baseline Wander

We modeled baseline wander as a linear combination of sinusoidal functions in the frequency range from 0 to 0.5 Hz. The amplitude and phase of each of the waveforms were chosen randomly to avoid deterministic coherence and to make each randomly generated signal unique. The upper limit of the frequency band was ten times larger than what is recommended for a high-pass filter for ST segment analysis. This fact in combination with very low SNR levels of up to −10 dB made this artifact a challenging one and allowed for performance ranking among the removal techniques [33, 34].

Mathematically speaking, the model was defined as follows:

(1) $$\begin{array}{c} b\left(\;t\;\right)=C\cdot \overset{K}{\underset{k=0}{\sum }}a_{k}\cdot \cos\left(\;2\pi \cdot k\cdot \Delta f\cdot t+\phi_{k}\;\right) \end{array}$$

with Δ*f* = *f*_*s*_/*N* = 0.01 Hz where *f*_*s*_ = 512 Hz was the sampling frequency of the signal and *N* = 51200 was the total number of samples in the signal. Additionally, *K* = *f*_*c*_/*f*_*s*_ = 50 was the number of sinusoidal functions that could be placed within the chosen spectrum of the baseline artifact according to the spectral resolution Δ*f*. The constant *a*_*k*_ was a random number from the uniform distribution [0; 1] and *ϕ*_*k*_ was a random phase in the interval [0; 2*π*). For each ECG signal *x*(*t*), we repeated the experiment 100 times generating a new realization of *b*(*t*) in each repetition. The scaling factor *C* was used to increase the total power of the artifact and modify the signal-to-noise ratio (SNR) in the experiment. The series of different SNR were chosen from the set [−10 dB, −3 dB, 0 dB, +3 dB, +10 dB, +20 dB].

The baseline is then added to the simulated ECG signal:

(2) $$\begin{array}{c} y\left(\;t\;\right)=x\left(\;t\;\right)+b\left(\;t\;\right). \end{array}$$

**Figure 3:**
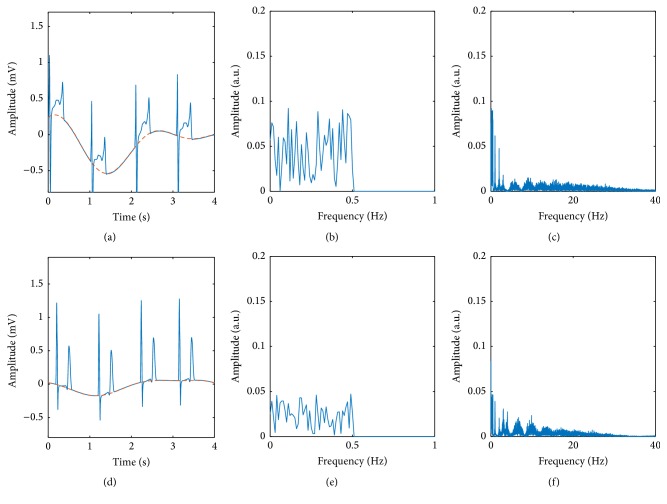
(a) Exemplary ECG signal corrupted by an arbitrary realization of the baseline wander model. The baseline wander artifact can be seen indicated by the red dashed line. An SNR of −3 dB was chosen for this example. (b) Frequency spectrum corresponding to the baseline wander artifact presented in (a). (c) Frequency spectrum corresponding to the signal (ECG plus baseline wander) displayed in (a). (d) A different example of an ECG signal corrupted by another realization of the baseline wander model. An SNR of +3 dB was chosen for this example. (e) Frequency spectrum corresponding to the baseline wander artifact presented in (d). (f) Frequency spectrum corresponding to the signal (ECG plus baseline wander) displayed in (d).

Again, *x*(*t*) is the reference ECG signal, while *y*(*t*) is the corrupted one. In this study, we created 756 simulations, 12 ECG channels per simulation, 100 realizations of the baseline wander model per channel, and six SNR levels of each baseline wander realization. Thus, a total of 5.508 million signals were used to evaluate the different baseline wander removal techniques including an extensive variety of scenarios.  Figure 3 shows two examples on how the baseline wander corrupted ECG signals and their spectra can look like depending on the different parameters.

#### 2.2.2. Signal Processing Workflow

**Figure 4:**
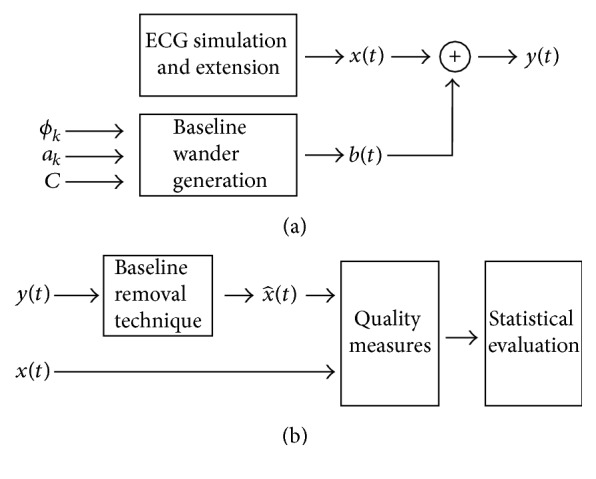
Signal processing workflow used in this study. (a) Creation the ECG and baseline wander artifact and the superposition to combine them. (b) The simulated and extended ECG is added to the baseline wander artifact to create the corrupted signal. The five baseline removal techniques are then applied to the corrupted signal to reconstruct the original one. To evaluate filtering performance, four different criteria are applied. At the end, the results are statistically analyzed to determine the best filtering method.

*y*(*t*) was filtered using five different baseline wander removal techniques. After filtering, the reconstructed signal $\hat{x}(t)$ was compared with the original *x*(*t*). The reconstruction was evaluated with respect to three quality criteria and the computation time.  Figure 4 shows a flow diagram of the signal processing algorithm. The components of the flow diagram will be explained in detail in the next sections.  Figure 4 shows the processing workflow created for this study.

#### 2.2.3. Baseline Removal Methods

We compared five state-of-the-art filtering techniques used regularly in literature: Butterworth high-pass filter [34], moving median and subtraction [35], spline approximation and subtraction [36], wavelet-based baseline cancellation [37], and wavelet-based high-pass filtering [38]. The filter parameters were chosen properly to be able to remove the given artifact from the ECG signal. A brief introduction to each method is given in the following sections.

#### 2.2.4. Butterworth High-Pass Filter

The first method has the property of being simple, easy to implement, and applicable in many scenarios. Therefore, a classic Butterworth high-pass filter with a total order of four and a cut-off frequency of 0.5 Hz was chosen. Actually, the transfer function of the filter had an order of 2 but the filtering process was performed in forward and reverse direction creating a zero-phase filtered signal and a resulting order of four [34].

#### 2.2.5. Moving Median and Subtraction

The second method has the goal of estimating the baseline wander using a concatenation of two moving median filters and subtracting that estimate from the corrupted signal. The moving median is based on the same principle as the moving average, but, instead of the mean, the median within a moving window of a given length is calculated. This filter benefits from the assumption that baseline wander and ECG signal have different amplitude distributions within the moving windows. The filter is nonlinear making its behavior more complex [35].

The removal technique started with a window length of 400 ms corresponding to the QT interval. Since windows of this short duration can deliver an estimation that is a mixture of true ECG signal and baseline, a second moving median with a window of a longer length was applied after the first estimation. We chose the second window to be 2 s long. By doing so, the complete frequency band of the artifact was included in the baseline estimation.

#### 2.2.6. Spline Approximation and Subtraction

The idea behind this method was to detect the center of the PQ interval in every beat and to interpolate those points to create an estimate of the baseline wander. This technique assumes that the PQ interval corresponds to the isoline of the ECG so that a nonzero signal in this interval must be due to baseline wander. Cubic splines were then used to connect the PQ center points and reconstruct the artifact. Finally, the estimated baseline wander was subtracted from the corrupted signal to reconstruct the original ECG [36].

#### 2.2.7. Wavelet-Based Baseline Cancellation

In this method, the signal was decomposed using the discrete wavelet transform (DWT) and the approximation coefficients at the lowest frequency band were set to zero with the aim of fully cancelling baseline wander. The filtered ECG was then reconstructed by synthesizing the modified coefficients. The decomposition level *L* for the DWT was chosen such that the approximation coefficients in that level corresponded to the frequency band where the artifact was located [37, 39]. Since in every decomposition level the ECG signal was downsampled by a factor of two, its Nyquist frequency was also halved. Thus, the necessary decomposition level to achieve a cut-off frequency *f*_*c*_ depending of the sampling frequency *f*_*s*_ can be given by

(3) fc=fs2L+1⟹L=log2⁡fsfc−1=9.

The wavelet used for this procedure was Daubechies 8 that has a compact support of 16 samples and is characterized by 8 vanishing moments.

#### 2.2.8. Wavelet-Based High-Pass Filtering

The principle behind this method is very similar to the wavelet-based baseline cancellation, but a high-pass filtering is used on the approximation coefficients instead of setting them to zero. This is somewhat comparable to a soft threshold on the approximation coefficients. For the wavelet decomposition, the Vaidyanathan-Hoang wavelet was used in accordance with [38]. For the high-pass filtering, an infinite impulse response filter of order one and a cut-off frequency of 0.5 Hz was used. The reconstructed ECG was synthesized from the filtered wavelet coefficients.

#### 2.2.9. Quality Criteria

Four performance indexes were chosen to evaluate the filters regarding the quality of reconstruction and the clinical applicability in terms of simplicity of the algorithm. The four evaluation criteria will be explained in detail.

As all filtered signals undergo a transient oscillation at their boundaries, we removed the first and final second (corresponding approximately to one beat) at the beginning and at the end of each signal from the evaluation analysis.

#### 2.2.10. Correlation Coefficient

With the aim of quantifying impairment in the morphology of the reconstructed signals, we used the correlation coefficient. It is independent from scaling or offsetting the signals and focuses on the matching form of original and reconstructed waveforms. Mathematically speaking, the correlation coefficient between the original signal *x*(*t*) and the reconstructed one $\hat{x}(t)$ is given by

(4) $$\begin{array}{c} CC\left\{\;x\left(\;t\;\right),\hat{x}\left(\;t\;\right)\;\right\}=\frac{E\left\{\left(x\left(t\right)-\mu_{x}\right)\left(\hat{x}\left(t\right)-\mu_{\hat{x}}\right)\right\}}{\sigma_{x}\sigma_{\hat{x}}}, \end{array}$$

where *E*{·} denotes the expected value operator, *μ*_*x*_ is the expected value of *x*(*t*), and *σ*_*x*_ is its standard deviation.

#### 2.2.11. *l*_operator

The *l*_operator is a measurement of similarity that is actually based on the Euclidian distance between the two signals. Mathematically speaking, it is defined as follows:

(5) $$\begin{array}{c} l\_operator\left\{\;x\left(\;t\;\right),\hat{x}\left(\;t\;\right)\;\right\}=1-\frac{E\left\{{\left(x\left(t\right)-\hat{x}\left(t\right)\right)}^{2}\right\}}{E\left\{x^{2}\left(t\right)\right\}+E\left\{{\hat{x}}^{2}\left(t\right)\right\}}. \end{array}$$

The *l*_operator has the advantage of delivering values in the interval [−1, +1]. It is equal to +1 only if the two signals *x*(*t*) and $\hat{x}(t)$ are perfectly equal. In contrast to the correlation coefficient, the *l*_operator is sensitive to offsetting and scaling of any of the two signals. It was originally introduced by our group to quantify changes in the morphology of the T wave [40].

#### 2.2.12. Deviation of the ST Change

For the clinical diagnosis of ST changes, thresholds for deviations in the J point of the ECG have been recommended [41]. Depending on the ECG lead, patient gender, and age, the ST changes can be diagnosed if a decrease of as low as 50 *μ*V is observed. Thus, it is necessary to preserve the original J point value as unchanged as possible after the baseline wander artifact has been removed. However, it is not always trivial to detect this point using automatic signal processing algorithms [42]. In order to avoid possible detection errors and incorrect results, we defined a new point inside the ST segment, the* K point *(KP), that can be used in same manner. The KP was defined as the time step for which the envelope (absolute value) among all ECG leads in a simulation is minimal. Mathematically speaking, it is given by

(6) $$\begin{array}{c} KP=\underset{t_{\mathrm{ST}}}{\min}\left\{\;\underset{i}{\max}\left|\;x_{i}\left(\;t_{ST}\;\right)\;\right|\;\right\}. \end{array}$$

In this case, the signal *x*_*i*_(*t*) denotes the ECG recorded from one of the 12 standard leads (*i* = 1,2, 3,…, 12). The time interval *t*_ST_ corresponds to the duration of the ST segment. A complete description of the KP can be found in [21]. The deviation in KP is then defined as

(7) $$\begin{array}{c} \Delta KP=KP_{filtered}-KP_{original}. \end{array}$$

#### 2.2.13. Computation Time

In a clinical environment, the computation time plays an important role if a fast diagnosis should be delivered by the physician. Faster computation times also correlate with simpler algorithms easier to implement for portable or stationary clinical devices. Thus, the computation time needed to process each signal was the fourth performance index. The computer used to run the calculations has a 2.4 GHz Intel Xeon E5645 processor with 12 cores and 64 GB of RAM running MacOS and Matlab 2016a.

### 2.3. Statistical Analysis

For the statistical analysis, we compared the performance of the five baseline removal techniques applied on the complete data set with respect to the four quality criteria mentioned previously. In addition, we also quantified all performance indexes for the case that no filter was applied. The idea behind this comparison is to find a “clear winner” among the filtering techniques that can be applied in many different scenarios.

We hypothesize that the best baseline removal technique for a given performance index was the one with the best median. This hypothesis was accepted if the statistical distribution of that performance index is significantly higher than all other methods. For statistical significance testing, we used the Wilcoxon signed rank test and a level of significance (*p* value) of 5%. This statistical test is a paired unparametric test commonly used in the field of signal processing [43–45].

When comparing a candidate for best performing filter to all other filters, a total of five *p* values are calculated. However, only the lowest *p* value is considered for further analysis. If that *p* value is below the level of significance, the filtering technique is leveled as the “clear winner.”

## 3. Results

### 3.1. Performance Evaluation Based on Quality Criteria

**Figure 5:**
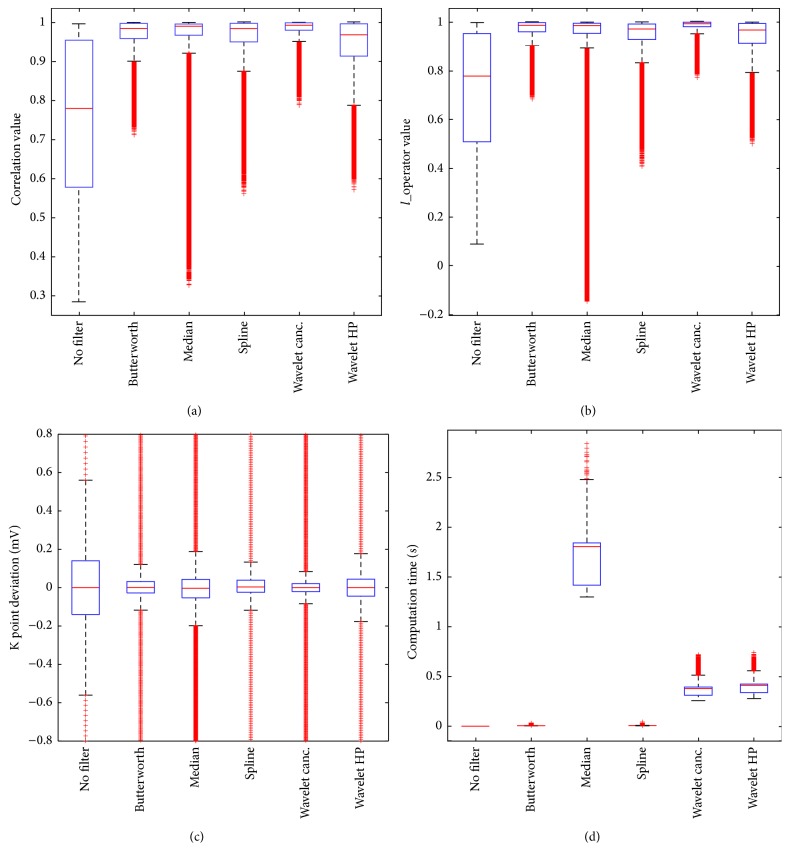
Boxplots displaying the results of performance evaluation of the filtering techniques. (a) Correlation coefficient between original and filtered signal, (b)* l*_operator between original and filtered signal, and (c) KP deviation between original and filtered signal. For visualization purposes, some outliers are not displayed in the figure. (d) Computation time needed to filter each signal.

We present the results of the simulation study in the form of boxplots and a summarizing table. Figures 5(a)–5(d) show the boxplots corresponding to the performance index values for all signals.  Table 1 contains the median and interquartile range of each quality criterion and filtering technique. The *p* value displayed in the last column of Table 1 is the highest one among all five comparisons.

According to the chosen evaluation scheme, the method that best maintained the original ECG morphology (highest correlation coefficient) was the wavelet-based baseline cancellation with a median and interquartile range (MED ± IQR) of 0.993 ± 0.019. Statistical testing proved that this method was indeed a clear winner with a *p* value <10^−6^. Second, the method that restores the signal closest to the original one (highest* l*_operator) was also the wavelet-based cancellation with a MED ± IQR of 0.993 ± 0.018 and a significant *p* value <10^−6^. Third, the method for which the ST changes undergo the lowest modifications (KP deviation) was again wavelet-based baseline cancellation with a MED ± IQR of 0.00 m*V* ± 0.042 m*V* and *p* value <10^−6^. Statistical testing proved that this method was again a clear winner in this category. Last but not least, the fastest method (computation time) was the Butterworth filter with a MED ± IQR of 0.006 s ± 0.001 s. Again, statistical testing proved this method to be speedier than all others with a *p* value <10^−6^.

**Figure 6:**
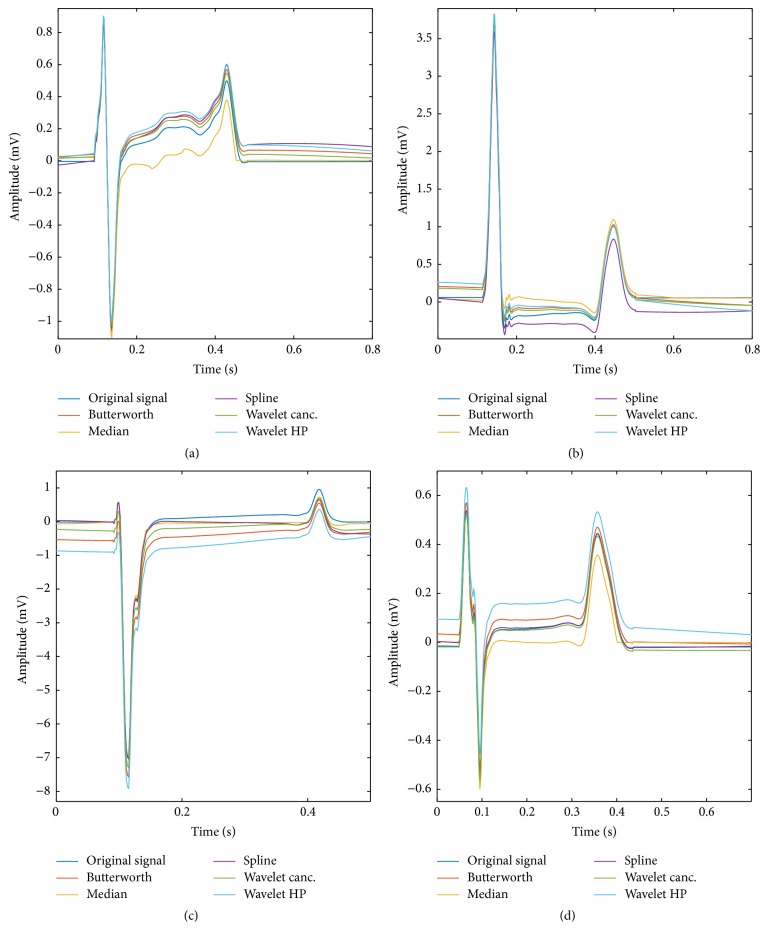
Four particular examples showing how the filtered signals compare to the original ones. (a) Filtering results for a signal that came from the ECG lead I in the first torso model and had an SNR of 0 dB. (b) Filtering results for a signal that came from the ECG lead II in the third torso model and had an SNR of +3 dB. (c) Filtering results for a signal that came from the ECG lead V2 in the second torso model and had an SNR of −10 dB. (d) Filtering results for a signal that came from the ECG lead aVL in the third torso model and had an SNR of −3 dB.

Finally, some particular examples showing how the filtered signals compare to the original ones are shown in Figure 6.

## 4. Discussion

In the simulation study, we saw that the wavelet-based baseline cancellation was the best performing method achieving the highest median and lowest IQR for the correlation coefficient,* l*_operator, and KP deviation. This result proves this filter to be not only the most accurate one (MED was best) but also the most robust (IQR was lowest). The reason for this is probably due to the properties of the chosen wavelet and the relatively high decomposition level. The combination of these two features matches precisely the time and frequency domain properties of the artifact. In the time domain, the chosen wavelet has 8 vanishing moments. This means that all polynomials up to a degree of 8 do not correlate with this wavelet and are represented in the approximation coefficients. Since our baseline wander model is a superposition of sinusoidal functions and they can be locally approximated by a Taylor polynomial of lower degree, it is plausible to believe that the approximation coefficients in the wavelet transform represent a large portion of the artifact. In addition, by a decomposition level of 9, the resulting wavelet filter has a very high order and is able to sharply split the spectrum of the corrupted ECG signal at precisely 0.5 Hz. By setting the resulting approximation coefficients to zero, the artifact is cancelled out almost entirely. However, this argumentation is only valid, if the true ECG signal cannot be locally approximated by a polynomial of order 8, or at least not as good as the artifact. Furthermore, the most relevant spectral components of the pure ECG signal must be above 0.5 Hz. Otherwise, the wavelet decomposition would have combined low frequency ECG components and the artifact into the approximation coefficients leading to a partial cancellation of the true ECG. The results observed here are also in accordance with what has been reported in literature [12].

A similar performance in terms of correlation coefficient and* l*_operator was delivered by the median filter. However, the KP deviations had a larger IQR of 96.7 *μ*V making this method less robust with respect to changes in the ST segment and decrease the certainty of a clinical diagnosis [41]. In addition, the sorting algorithm needed in this technique is computationally intensive leading to long processing times while filtering the signal. This could become a major drawback in clinical applications. In contrast to it, the fastest method was the Butterworth filter taking around 5.9 ms to process the corrupted ECG. The speedy computation can be achieved because the chosen filter order of two (four in the zero-phase implementation) was relatively small. The filtering process can be performed very fast as a linear combination of no more than four input and output signal values for each the forward and the backward filtering process. Moreover, this technique is capable of reconstructing the original ECG with a median correlation coefficient of 0.985 and a median* l*_operator of 0.986, which ranks this method in the third place. More importantly, the Butterworth filter was the second best performing filter with respect to KP deviations (1 *μV* ± 59 *μV*). This is a surprising result for such a simple filter. In particular for medical applications that require fast but still accurate signal processing algorithms, this is the method we would recommend. If a zero-phase implementation is not required, the Butterworth filter can also be approximated as a finite impulse response filter especially suited for real time applications.

The results demonstrated that even though there were small differences among the methods, they were all good performers in terms of correlation coefficient,* l*_operator, and KP deviations. However, such good results are not necessarily to be expected in real-life applications because a priori knowledge about the time and frequency domain properties of the baseline wander model was used to manually choose filter parameters. The results are probably overfitted but since all filters were adapted to match the baseline wander model, we assume that the comparability among them should still be given. Thus, the ranking of the filter techniques should remain in practice.

We also reaffirmed that baseline wander can indeed strongly affect an ECG. The nonfiltered signal had a median correlation coefficient of 0.779 and an IQR of the KP deviation of 280.2 mV. Thus, removing the baseline wander becomes mandatory to allow any further processing of the ECG. We also found that even though all methods deliver an improvement, the KP deviation after filtering had an IQR of at least 41.9 *μ*V in every case. This means that none of the filtering techniques is capable of reconstructing the ST segment to its exact original shape. Hence, in clinical applications dealing with the diagnosis of ischemia, the assessment of the filtered ECG signal has to be carried out very carefully because the observed ST change might not be the true one.

### 4.1. Limitations and Outlook

This study ranks popular baseline wander removal techniques using a reference ECG signal that is free of artifacts but exhibits all the properties of an ischemic ECG. The simulation of realistic ECGs is a challenging task because of the complexity of the underlying electrophysiological behavior reproduced by the multiscale model. This model is governed by a large variety of coupled differential equations that need to be correctly parametrized first [21]. In particular, the recreation of the T wave is difficult because this wave arises from the heterogeneities in the repolarization of the ventricles and, thus, the APs have to be adapted to each region of the heart [17]. To overcome this problem, it could be stated that the replication of a reference beat with ST change from a real recording would be a simpler way of performing the study. However, this approach has the drawback that it cannot be assured that the reference beat is completely free of artifacts and should not be used as a golden truth. Even if an ECG looks clean to the naked eye, little low frequency perturbations or a small DC offset is always present locally. Thus, it becomes difficult to quantify changes in morphology because the filter would modify not only the signal but also the artifacts previously present in it. In addition, annotations carried out by a trained physician would be necessary to ensure the validity of such a database. Under those conditions, it is impractical to create a data set with the same size and diversity as the one presented here. A reduced number of signals would limit the generalization of the filter ranking. A further idea would be the use of an ECG synthesizer with adjustable ST change [46, 47]. Yet, this approach has the drawback that the user can never be sure that the synthetized ECG is realistic enough to recreate real ST changes as they arise from ischemia. For these reasons, we believe that the in silico study based on a ischemic membrane model delivers a reference signal that is free of artifacts but still meets the requirements for a realistic ischemic ECG and allows us to generate a large number of scenarios.

Even though the total amount of signals used in this study was large, there were only three different geometries and one electrophysiological model (Ten Tusscher) used to simulate ischemia [26]. It would be interesting to use other electrophysiological models with other patient geometries having also different fiber orientations in the heart to generate a more heterogeneous data set. In addition, the atrial activity characterized by the P wave can also be included in a future simulation. The P wave would affect not only the time domain properties of the synthesized ECG but also its spectral and statistical features. They pose a further challenge to the baseline removal methods.

A more sophisticated baseline wander model would also be of interest. It is well-known that respiration cannot only lead to a floating baseline but it can also modulate the ECG signal [48, 49]. Thus, a baseline wander model in which the artifact affects both, the isoline and the amplitude of the ECG, should be considered in a future research project. Additionally, other spectral properties could also be included in the artifact. Increasing its frequency band up to 3 Hz, for example, would make the filtering problem more challenging. However, in those cases, the artifact overlaps in the time and frequency domain with the T wave and the ST segment. This makes the reconstruction of the original signal very difficult and a diagnosis is probably no longer possible. Other studies have investigated how to eliminate baseline wander with spectral content up to 0.8 Hz [15, 38]. In those cases, the spectral content of the artifact starts to overlap with the dominant frequency of the heart rate in resting conditions making this scenario particularly difficult. Thus, this kind of removal techniques should only be performed with filters having linear phase. This was not the case for the majority of the filters used here and, for that reason, we chose our upper frequency at 0.5 Hz.

The filter parameters used in this work were chosen in a heuristic manner with the intention of having a good performance for the well-known artifact. However, they were not computationally optimized to deliver the best possible results. For example, the order of the Butterworth filter, the length windows of the median filters, or the wavelet used for the decomposition could all be further optimized to achieve even better results. This optimization process could also be included in a future work together with other more less common baseline removal techniques such as the empirical mode decomposition, the blind source separation, or a Gaussian filter adapted to remove the known spectrum of the artifact [50–52].

The use of the KP instead of the J point to evaluate the ST change deviations was a strategic decision in order to allow automatized quantification of performance. Allowing a trained physician to annotate the J point and perform the study with the annotations might deliver different results. However, by the large amount of signals simulated, a manual annotation becomes unpractical. In any case, we were consistent using the same definition for all filtering techniques and allowing comparability among them.

Last but not least, we quantified the changes in the ST segment caused by filtering measuring the deviation in KP. Yet, the true clinical impact of the filters on the diagnosis of an ischemia was not studied. We did not count how many ischemia cases would have been missed because of the filtering process. This question is not easily answered because the identification of an ST change can be compromised by other factors besides the filters. Those factors are, for example, a large variety of silent ischemia, the number of electrodes used in the recording, or the placement of the electrodes on the chest of the patient [22]. This is the reason why the sensitivity of the ischemia detection, even if a trained physician is looking at the ECG, can be as low as 45% [53]. We investigated this limitation in a previous simulation study obtaining similar results as the ones observed in clinical studies. We also proved that the sensitivity of the ischemia diagnosis is strongly dependent on the chosen threshold defining the ST change [21]. In any case, it would be interesting for a future project to analyze how these factors play together with the baseline removal techniques and how they all affect the ischemia detection. For now, it is certain that the better the filters perform on the corrupted ECG, the higher the chance of a physician making the right decision is.

## 5. Conclusion

In this study, we addressed the question of the best suited technique for baseline removal without compromising ST changes in the ischemic ECG. For this purpose, a large simulation study with 5.508 million signals was carried out. The best performing filter with respect to quality of the reconstruction turned out to be the wavelet-based baseline cancellation. However, for medical applications, the Butterworth high-pass filter is the better choice because it is computationally fast and almost as accurate. In addition, all the methods tested proved to be better than leaving baseline wander unfiltered. It was also shown that none of methods was capable of reconstructing the original ECG without modifying the ST segment, so the user has to be always very careful when diagnosing an ST change. In future, other baseline wander models including nonlinear behavior and higher frequency baseline wander could be used to test the methods in more challenging scenarios.

## Figures and Tables

**Table 1:** Summary of the results obtained for the performance evaluation among the filters. The values are given as MED ± IQR. The *p* values display the statistical significance of the best performing filter (bold numbers) in each category.

Filter/indexes	Correlation	*l*_operator	KP deviation [mV]	Computation time [s]
No filter	0.7794 ± 0.3764	0.7797 ± 0.4452	0.0000 ± 0.2802	0 ± 0
Butterworth	0.9851 ± 0.0392	0.9859 ± 0.0372	0.0010 ± 0.0595	0.0059 ± 0.0434
Median	0.9904 ± 0.0299	0.9872 ± 0.0413	−0.0033 ± 0.0967	1.8464 ± 0.0434
Spline	0.9813 ± 0.0486	0.9716 ± 0.0629	0.0037 ± 0.0628	0.0074 ± 0.0434
Wavelet cancellation	0.9928 ± 0.0194	0.9933 ± 0.0184	0.0000 ± 0.0419	0.3892 ± 0.0434
Wavelet high-pass	0.9672 ± 0.0827	0.9689 ± 0.0816	0.0000 ± 0.0885	0.4206 ± 0.0189

*p* values	<10^−6^	<10^−6^	<10^−6^	<10^−6^
